# Meningitis gone viral: description of the echovirus wave 2013 in Germany

**DOI:** 10.1186/s12879-019-4635-6

**Published:** 2019-11-29

**Authors:** Jonas Graf, Christian J. Hartmann, Helmar C. Lehmann, Carolin Otto, Ortwin Adams, Michael Karenfort, Christian Schneider, Klemens Ruprecht, Hans Martin Bosse, Sabine Diedrich, Sindy Böttcher, Alfons Schnitzler, Hans-Peter Hartung, Orhan Aktas, Philipp Albrecht

**Affiliations:** 1 Department of Neurology, University Hospital, Medical Faculty Heinrich-Heine University, Moorenstraße 5, 40225 Düsseldorf, Germany; 2 Department of Neurology, Center for Movement Disorders and Neuromodulation, Medical Faculty, University Hospital, Medical Faculty, Heinrich-Heine University, Düsseldorf, Germany; 3 Institute of Clinical Neuroscience and Medical Psychology, Medical Faculty, Heinrich-Heine University, Düsseldorf, Germany; 4 Department of Neurology, University Hospital of Cologne, Cologne, Germany; 5 Department of Neurology, Charité – Universitätsmedizin Berlin, corporate member of Freie Universität Berlin, Humboldt-Universität zu Berlin, Berlin Institute of Health, Berlin, Germany; 6 Institute of Virology, University Hospital, Heinrich-Heine University, Düsseldorf, Germany; 7 Department of General Pediatrics, Neonatology and Pediatric Cardiology, University Hospital, Heinrich-Heine University, Düsseldorf, Germany; 8 FG 15 Nationales Referenzzentrum für Poliomyelitis und Enteroviren, Robert Koch Institut, Berlin, Germany

**Keywords:** Meningitis, Echovirus, Epidemic, Surveillance

## Abstract

**Background:**

Aseptic meningitis epidemics may pose various health care challenges.

**Methods:**

We describe the German enterovirus meningitis epidemics in the university hospital centers of Düsseldorf, Cologne and Berlin between January 1st and December 31st, 2013 in order to scrutinize clinical differences from other aseptic meningitis cases.

**Results:**

A total of 72 enterovirus (EV-positive) meningitis cases were detected in our multicenter cohort, corresponding to 5.8% of all EV-positive cases which were voluntarily reported within the National Enterovirus surveillance (EVSurv, based on investigation of patients with suspected aseptic meningitis/encephalitis and/or acute flaccid paralysis) by physicians within this period of time. Among these 72 patients, 38 (52.8%) were enterovirus positive and typed as echovirus (18 pediatric and 20 adult cases, median age 18.5 years; echovirus 18 (1), echovirus 2 (1), echovirus 30 (31), echovirus 33 (1), echovirus 9 (4)). At the same time, 45 aseptic meningitis cases in our cohort were excluded to be due to enteroviral infection (EV-negative). Three EV-negative patients were tested positive for varicella zoster virus (VZV) and 1 EV-negative patient for herpes simplex virus 2. Hospitalization was significantly longer in EV-negative cases. Cerebrospinal fluid analysis did not reveal significant differences between the two groups. After discharge, EV-meningitis resulted in significant burden of sick leave in our pediatric cohort as parents had to care for the children at home.

**Conclusions:**

Voluntary syndromic surveillance, such as provided by the EVSurv in our study may be a valuable tool for epidemiological research. Our analyses suggest that EV-positive meningitis predominantly affects younger patients and may be associated with a rather benign clinical course, compared to EV-negative cases.

## Background

Periodic aseptic meningitis epidemics can be a challenge in patient- and health care. A large retrospective analysis of a US-American cohort revealed that in 21% of cases the etiology of aseptic meningitis remains unknown [1]. Aseptic meningitis is defined [2] by an inflammation of the leptomeninx in which the causative agent cannot be identified by cerebrospinal fluid culture [3]. Viruses are the most common causes of this disease [1, 3]. Viral meningitides are predominantly caused by enteroviruses [4], which belong to the picornaviridae consisting of species A-D. The main route of infection is fecal-oral, but infestation of the respiratory tract and a droplet infection are also possible. In previous studies, viral meningitis in adults was rather associated with herpes simplex and West Nile virus, whereas children were more likely to be tested positive for enterovirus (EV) [5]. Therefore, multiple studies have been conducted in order to better understand this phenomenon: A Danish nation-wide prospective observational study between 1st of January 2015 and 30th of June 2016 revealed an unfavorable outcome of viral meningitis in 17% of all patients [6]. According to a UK study, the infection rates of viral meningitis are mainly driven by an EV predominance of echovirus 30 [7].

EV meningitis epidemics in Shandong (People’s Republic of China, 2014 [8]) and Finland (2009 and 2010 [9]) and the clinical pattern of viral central nervous system (CNS) infections in Italy [10] have previously been characterized: EV-positive patients presented with fever, nausea and vomiting, were most likely to be children, and had no clear gender predominance.

The treatment is symptomatic, employing analgetic drugs and antipyretic therapy to control body temperature. Pleconaril has been considered as potential specific treatment for EV-associated meningitis. However, it was not approved, given its just modest efficacy and considerable side-effect and interaction profile [11, 12]. In particularly severe cases, administration of immunoglobulins may positively influence the course of the disease [13].

Cerebrospinal fluid (CSF) and clinical features of EV-positive meningitis patients in Germany [14] and the differences in adult and pediatric EV-positive meningitis patients in Switzerland [15] have already been analyzed, but there is still a paucity of data describing the differences in EV-positive meningitis and EV-negative meningitis patients.

## Methods

We conducted a retrospective chart review study at the Departments of Neurology of the Heinrich-Heine University Düsseldorf, the University Hospital of Cologne, the Charité – Universitätsmedizin Berlin and the Department of General Pediatrics, Neonatology and Pediatric Cardiology of the Heinrich-Heine University Düsseldorf searching for all patients with aseptic CNS infection in 2013. The study was approved by the ethics committee, University of Düsseldorf (registry number 4423). We used ICD-10 codes to identify cases of interest. As such, priority was given to the ICD-10 keys A87 and G02 (Table 1). However, as patient data may not have been in the categories listed above due to less precise encryption despite manifest illness, a wider query was additionally performed to identify all patient data encoded as A85-A89 (Other viral encephalitis, not elsewhere classified; Unspecified viral encephalitis; Viral meningitis; Other viral infections of central nervous system, not elsewhere classified; Unspecified viral infection of central nervous system, Table 1) and G02–05 (Meningitis in other infectious and parasitic diseases classified elsewhere; Meningitis due to other and unspecified causes; Encephalitis, myelitis and encephalomyelitis; Encephalitis, myelitis and encephalomyelitis in diseases classified elsewhere, Table 1). Virological testing of CSF for enteroviruses was performed in a standardized manner by the National Reference Laboratory for Poliomyelitis and Enteroviruses at the Robert Koch Institute.

**Table 1:** List of ICD-10 codes utilized to identify patients with aseptic meningitis from the clinical databases of each hospital participating in this study

ICD-10	Description
A85	Other viral encephalitis, not elsewhere classified
A86	Unspecified viral encephalitis
A87	Viral meningitis
A88	Other viral infections of central nervous system, not elsewhere classified
A89	Unspecified viral infection of central nervous system
G02	Meningitis in other infectious and parasitic diseases classified elsewhere
G03	Meningitis due to other and unspecified causes
G04	Encephalitis, myelitis and encephalomyelitis
G05	Encephalitis, myelitis and encephalomyelitis in diseases classified elsewhere

### Case definition

After the above-mentioned identification of patient data, the patient records were individually evaluated to exclusively select cases of aseptic meningitis defined according to the Centers for Disease Control and Prevention (CDC) [16] as acute onset of meningeal symptoms, fever, and cerebrospinal fluid pleocytosis with bacteriologically sterile cultures.

### Analysis

The following criteria were investigated for further analysis: Patient age, time point of manifestation, prehospital time / duration of clinical manifestation before confirmation of aseptic meningitis by CSF analyses, results of CSF diagnostics (cell count, protein content), duration of inpatient stay, type of clinical restitution (complete restitution vs. persistence of residual symptoms), for children treated in Düsseldorf time of incapacity for work of parents. CSF cell count and protein content were measured according to the local laboratory standard (Düsseldorf: turbidimetric, benzethonium chloride method, cobas® 8000, C701, Fa. Roche Diagnostics, Mannheim for protein content, mechanized cell count, UF 1000i, Sysmex for cell count; Berlin: turbidimetric assay TPUC3, Roche/Hitachi cobas® c for protein content, Fuchs-Rosenthal method for cell count; Cologne: nephelometric assay for protein content, Fuchs-Rosenthal method for cell count).

In order to put this data in perspective, we performed a query of the German-wide database of the Robert Koch Institute (RKI; EVSurv) [17] of all EV-positive meningitis cases in 2013. Data was obtained in the context of the National Enterovirussurveillance, which is based on voluntary reporting and investigation of hospitalized patients with suspected aseptic meningitis/encephalitis and/or acute flaccid paralysis. All samples were tested at the RKI using RT-nested PCR with the primers targeting the 5′NCR gene, as previously described [18]. All PCR products were sequenced and - based on the resulting EV species - tested with species-specific PCR assays in the VP1 region for typing as recently described [19–21].

Statistical analyses were performed as indicated using SPSS Version 20 (IBM Corp. NY, USA); non-parametric testing was performed since all investigated variables were non-normally distributed (Shapiro-Wilk-test); *p* values < 0.05 were considered significant. If not specified otherwise, data are provided as median (25th; 75th percentile). Furthermore, a Chi-Square-test was performed in order to compare adults with children and a Spearman’s correlation was performed in order to explore predictors of hospitalization. Adjusted *p*-values (adj. p) were calculated using Bonferroni correction, values below 0.05 were considered significant. To enhance readability of the results section, significant values are provided in three categories: < 0.05, < 0.01, and < 0.001, respectively. Finally, a multivariate, stepwise linear regression was performed in order to extract variables of potential predictive value for the time of hospitalization.

## Results

### Results of the retrospective analysis in Düsseldorf, Cologne and Berlin 2013.

**Fig. 1:**
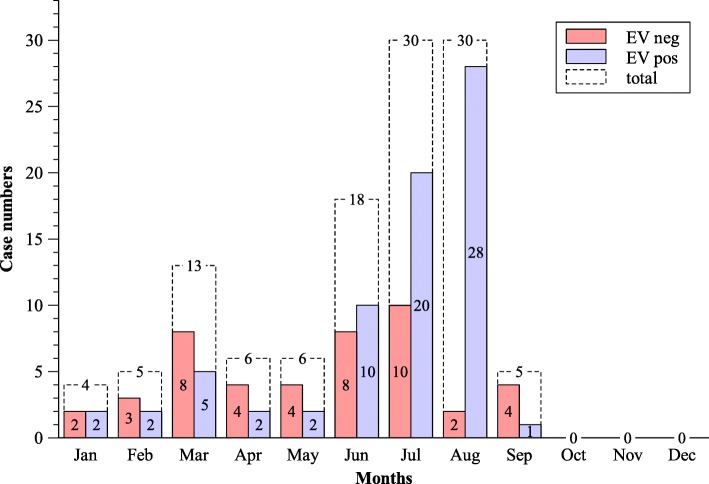
Monthly distribution of meningitis cases in 2013 (Düsseldorf, Cologne, Berlin). These data were obtained from database query. Enterovirus (EV)-positive cases peaked from July to August in 2013

We identified 72 EV-positive cases (31 females, 41 males) with a median age of 15 (3.25; 32.75) years. Among these 72 patients, 38 (52.8%) were echovirus-positive (18 pediatric and 20 adult cases, median age 18.5 (5.25; 31.25) years; echovirus 18 (1), echovirus 2 (1), echovirus 30 (31), echovirus 33 (1), echovirus 9 (4)), 1 patient was enterovirus 71 (EV-A71)-positive, 1 patient was coxsackie A9-positive, 1 patient was enterovirus B-positive and the specific enterovirus species of the remaining 31 EV-positive cases remained unknown or were not further typed, as not enough CSF was available for further analysis. EV-positive meningitis cases peaked in July/August (Fig. 1). Furthermore, we identified 45 enterovirus-negative cases (16 females, 29 males, median age 36 (28; 48.5) years). Among EV-negative cases, three were related to varicella zoster infection, one to herpes simplex 2 and no virus could be identified in the other patients.

**Fig. 2:**
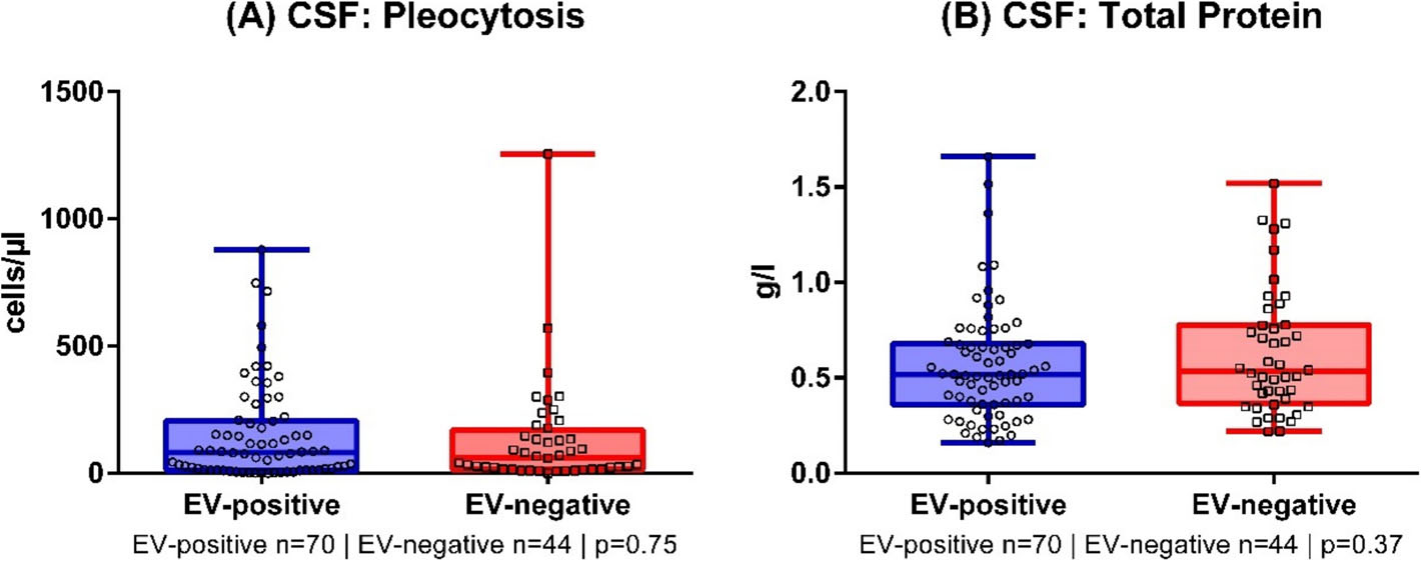
Cerebrospinal fluid analysis of enterovirus (EV)-positive and EV-negative patients. No significant difference in cell count (**a**) and total protein (**b**). CSF = cerebrospinal fluid. Mann-Whitney *U* test

Analysis of the CSF parameters cell count (EV-positive: 81 (12; 205) cells/μl, EV negative 67 (17.5; 185.25) cells/μl, Fig. 2a) and total protein (EV-positive 0.52 (0.35; 0.68) g/l, EV-negative 0.53 (0.36, 0.78) g/l, Fig. 2b) in EV-positive and EV-negative patients revealed no significant difference (Mann-Whitney *U* test). 2 EV-positive and 1 EV-negative CSF samples could not be evaluated due to a blood contamination.

**Fig. 3:**
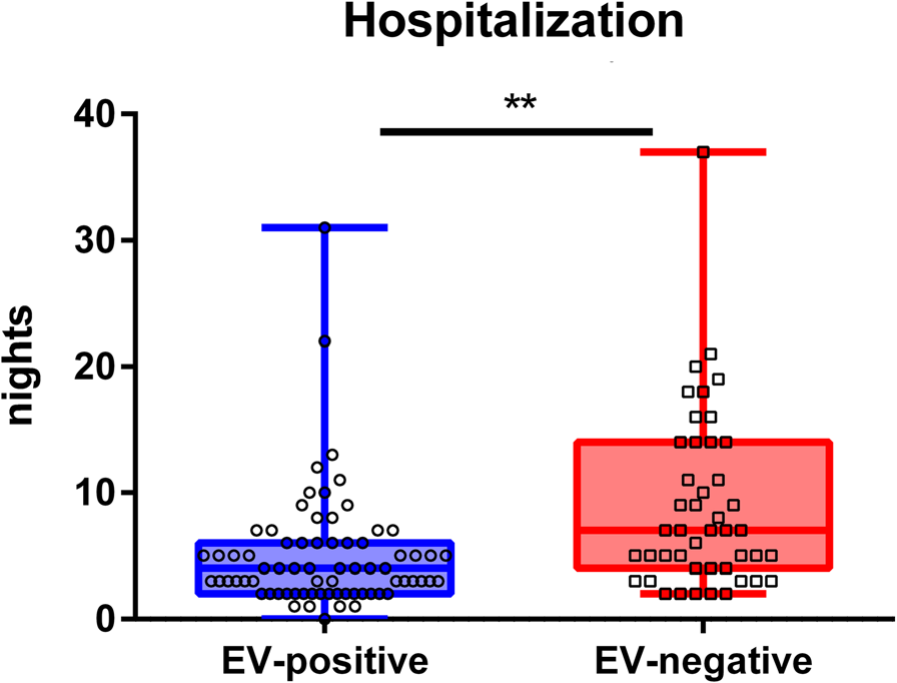
Hospitalization analysis of enterovirus (EV)-positive (*n* = 72) and EV-negative patients (*n* = 45). Hospitalization of EV-negative patients is significantly longer (*p* < 0.01). Mann-Whitney *U* test

Analysis of the number of nights spent in hospital by the patients revealed that hospitalization was significantly longer in EV-negative (6 (3; 13) nights) than in EV-positive cases (3 (1; 5) nights, adj. *p* < 0.01, Mann-Whitney *U* test, Fig. 3).

No fatal cases occurred. In children, parents reported of mild complaints after discharge like headache, backache and fatigue for 0 to 7 days (median 2 days; Düsseldorf cohort). After discharge, one of the parents had to care for the children at home (0 to 5 days, median 3 days; Düsseldorf cohort).

Analysis of gender (Chi-Square-test of EV-positive vs. EV-negative patients, adults vs. children and hospitalization periods) did not reveal relevant differences.

### Adults vs. children

A significantly higher ratio of children was found in the enterovirus-positive cohort, compared to the enterovirus negative group (38/72 vs. 4/45, Chi-Square-test, adj. *p* < 0.001). Children (both EV-positive and EV-negative) had a shorter period of hospitalization (adj. *p* < 0.001) and lower CSF protein levels than adults (adj. *p* < 0.001). There was no significant difference of CSF cell counts (Mann-Whitney *U* test, respectively).

An exclusive analysis of either adults or children did not reveal significant differences between EV-positive and EV-negative patients regarding age, duration of inpatient stay, CSF cell count and CSF total protein (Mann-Whitney *U* test, respectively). Numerical data of abovementioned comparisons are provided in Table 2.

**Table 2:** Distribution of age, hospitalization, CSF cell count, and CSF protein for EV negative, EV positive, and all patients (Total). All groups are further subdivided by age into adults (ADU), children (PED), and all (Total) patients. *N* = number of patients, 75th perc. = 75th percentile, 25th perc = 25th percentile

	EV negative			EV positive			Total		
	ADU	PED	Total	ADU	PED	Total	ADU	PED	Total
Age (years)									
*N*	*41*	*4*	*45*	*34*	*38*	*72*	*75*	*42*	*117*
Range	64	10	77	56	17	74	66	17	84
75th perc.	50	14.75	48.5	40	9	32.75	44	9	38
median	36	8	36	33.5	5	15	36	6	26
25th perc.	30	7.25	28	24	0	3.25	27	0.75	8
Hospitalization (nights)									
*N*	*41*	*4*	*45*	*34*	*38*	*72*	*75*	*42*	*117*
Range	35.00	5	35	31.00	9.00	31	36.00	9.00	36
75th perc.	13	5	13	6	3.25	5	10	3.25	7
median	6	2	6	4	2.00	3	5	2	4
25th perc.	3.5	1.25	3	1.75	1	1	2	1	2
CSF cell count (cells/μl)									
*N*	*40*	*4*	*44*	*34*	*36*	*70*	*74*	*40*	*114*
Range	1255	100	1255	744	877	877	1255	877	1255
75th perc.	202.25	129.75	185.25	314.75	142	205	275.75	132.25	196.5
median	61.5	100	67	114.5	24.5	81	88	34.5	78
25th perc.	15.25	44.75	17.50	40.5	6.75	12	23.75	9.5	15
CSF protein (g/l)									
*N*	*40*	*4*	*44*	*34*	*36*	*70*	*74*	*40*	*114*
Range	1.30	0.64	1.30	1.29	0.73	1.51	1.66	0.73	1.65
75th perc.	0.78	0.79	0.78	0.80	0.57	0.68	0.78	0.58	0.74
median	0.53	0.42	0.53	0.65	0.34	0.52	0.56	0.36	0.52
25th perc.	0.39	0.23	0.36	0.48	0.25	0.35	0.43	0.250	0.36

### Predictors of hospitalization periods

In general, the duration of inpatient stay correlated with age (Spearman’s Rho correlation coefficient 0.418, *p* < 0.001), CSF total protein (Spearman’s Rho correlation coefficient 0.319, *p* < 0.001), and the delay from symptom onset to lumbar puncture (Spearman’s Rho correlation coefficient 0.232, *p* = 0.023). For nominal variables, enterovirus status (η = − 0,32) correlated with the duration of inpatient stay. In contrast, echovirus status, gender, and location (Neurological center the patient was treated) did not show a relevant correlation with the duration of the inpatient stay (| η | < 0,3).

Finally, a multivariate, stepwise linear regression was performed using the abovementioned variables (age, CSF cell count, CSF protein, delay between symptom onset and spinal tap enterovirus status, echovirus status, gender, treating center (Berlin, Cologne, Duesseldorf)). A total of three variables (age, CSF protein, and Echovirus status) were kept, which accounted for 30% of the variance of the hospitalization period (adjusted *R*^*2*^ = 0.302, standardized Beta values: age = 0.354, CSF protein 0.247, and echovirus status − 0.169, respectively).

### Therapy

The cases diagnosed with varicella zoster virus and herpes simplex virus 2 received specific therapy. No child received specific therapy in Düsseldorf.

### RKI database query

An RKI database query (retrieved from https://evsurv.rki.de/) revealed a total of 3455 tested samples in 2013. 1242 of these cases were positive for EV, of which 672 cases were typed as echovirus 30. Therefore, our study includes 5.8% (72 of 1242 cases) of the reported EV-positive cases in Germany.

## Discussion

Enteroviruses are highly neurotropic and can manifest as meningitis, meningoencephalitis, poliomyelitis-like anterior myelitis, and Guillain-Barré syndrome [22, 23]. In our cohort, enteroviral infections were associated with meningitis. The prevalence of enteroviral meningitis is high worldwide (estimated 75,000 cases annually in the United States) [11], which makes this type of meningitis highly relevant to both caregivers and patients. We were able to show that the rate of infection peaks in the summer and early autumn months (June, July and August; Fig. 1). With age, the incidence of enteroviral meningitis decreases. Therefore, the incidence is highest in infants and toddlers [24], which was also the case in our cohort (Table 2).

The following findings and assumptions of our study are of significant interest, as they stress differences between EV-positive and EV-negative meningitis and may be of relevance for the treating physician: Overall, caregivers may expect shorter hospitalization times in EV-positive meningitis cases. Furthermore, routine CSF parameters that may already be determined in the emergency unit are not a sufficient tool to discriminate between EV-positive and EV-negative meningitis. When caregivers experience an unusual accumulation of aseptic meningitis cases in the summer and early autumn, patients should be tested for enterovirus infections and cases should be reported to the authorities. Contrary to previous studies regarding viral meningitis in general our data show that EV-positive meningitis is rather associated with a benign disease course.

In adult patients, the disease generally necessitates inpatient treatment for several days [25]. When children are affected, one parent may be incapacitated for a certain period of time to care for the child. In both cases, the disease may be associated with a temporary inability to work (either patient or parent). Because of the high number of cases per year, considerable costs arise for society due to the loss of work and the necessary resources for medical treatment [26], although meningitis caused by enteroviruses usually has a relatively benign course.

To the best of our knowledge, the economic burden for society due to EV-positive meningitis has not been determined so far; and our data also provide just a limited insight, since we analyzed the duration of inpatient stay but did not assess any further inability to work.

Our data indicate that the course of EV-positive meningitis is predominantly benign, and that hospitalization time was significantly shorter in EV-positive, compared to EV-negative cases. This was also the case, when we did not consider the above-mentioned meningitis cases that received specific antiviral therapy (varicella zoster virus, herpes simplex virus 2). Moreover, a higher ratio of affected children and young adults were found in EV-positive cases. This could be explained by affected parents of young, diseased children. Hence, earlier convalescence in EV-positive groups may be explained by differences of age between both groups rather than different courses of the disease in general. In our cohort, routine CSF analysis (pleocytosis, protein level) is not a useful tool to discriminate between EV-positive and negative cases, but CSF protein level may correlate with length of stay in hospital.

Despite the generally excellent outcome of aseptic meningitis, there are rare instances of complicated courses that may lead to persistent neurological disability or even death [27–30]. Strategies for the systematic containment of endemic diseases are focused on ensuring hygienic measures to prevent the spread of viruses, as some weeks after illness, virulent pathogens can still be excreted via the feces.

In agreement with previous studies, enterovirus infections were detected as the most common cause for an aseptic meningitis in our cohort, driven by a high prevalence of echoviruses (52,8%). Indeed, our study demonstrates that voluntary reporting of diseases such as in this case can be an effective tool to better understand epidemiological details of certain diseases:

The mean age of EV-positive patients in our centers was 15 (3.25; 32.75) years, which accurately fits to previous data of Shandong [8] and Finland [9] (Finland 2009 15 years 8 months, Finland 2010 17 years 6 months, Shandong 2014 children within 15 years of age). The mean age of EV-negative pediatric patients (8 (7.25; 14.75) years) in our cohort was quite similar to that in a large South Korean pediatric cohort (8.4 ± 5 years) [31].

## Conclusions

EV-positive epidemics are similar in terms of age and gender distribution and other factors worldwide. Overall, this entity remains a rather benign form of meningitis with a rather short length of stay in hospital, but may be associated with complicated courses that may lead to persistent neurological disability or even death. Routine CSF testing (pleocytosis, protein level) may not be suitable to distinguish EV-positive and EV-negative cases, but CSF protein level may correlate with hospitalization. Still, epidemics are a challenge for the health care system. Therefore, we recommend rigorous testing and reporting of aseptic meningitis cases. Within the National Enterovirussurveillance (EVSurv) all pediatric and neurological hospitals in Germany are offered free-of-charge enterovirus diagnostics in patients with suspected aseptic meningitis / encephalitis or acute flaccid paralysis. This health care concept is also well established in the US and led to a concise description of the disease burden [32].

## Data Availability

The datasets used and/or analysed during the current study are available from the corresponding author on reasonable request.

## Abbreviations

adj. p: Adjusted *p*-value
CDC: Centers for Disease Control and Prevention
CNS: Central nervous system
CSF: Cerebrospinal fluid
EV: Enterovirus
RKI: Robert Koch Institut
VZV: Varicella zoster virus
